# Aids to Memory

**Published:** 1888-08-11

**Authors:** 


### AIDS TO MEMORY.

Never having undergone Professor Loisette's treatment for the improvement of memory, we cannot say whether he uses mental or physical means; but to those of our readers who say they can remember nothing—it usually means only nothing in which they are not interested—we offer a few recipes for the improvement of the memory, which were very popular in mediæval times, as well as some information as to things our forefathers held to be injurious to that faculty. We have made no experiments with them, but on the whole they seem to be perfectly harmless, even if they are not the absolute specifics they claim to be.

In the first place there are the things calculated to injure the memory, which will perhaps be sought after by lovelorn damsels, truant schoolboys, and it may be repentant criminals. By all, therefore, who say or sing "Teach, oh teach me to forget," the following articles will be found useful, if we may believe Maistre Arnauld de Villeneuve, a highly-respectable practitioner of past times: Pork, and the flesh of cats (whether eaten as such, or disguised under the name of veal or rabbit, according to the traditional custom of vendors of pies), excessive drinking of water or wine—we quite agree with Maistre Arnauld anent this last indulgence; getting a chill when the body is warm, which lessens the natural heat of the body, and consequently, says our authority, corrupts and destroys the memory. We confess that we don't see the logic of this *consequently*, and should like to have the pathology of the matter explained to us more clearly; but doctors didn't think such information necessary in the "ages of faith." Again, excessive sleep injures the memory; and so, on the principle that extremes meet, does excessive wakefulness. With regard to the question of sleep, Villeneuve says further that sleeping too soon after eating is dangerous, because—here he gives us another *non sequitur*—the food has not descended to the bottom of the stomach; and sleeping with shoes on is also injurious. Finally, he states that great and sudden fear affects the memory, for which remark he had probably more foundation in fact and observation than for his condemnation of roast pussy-cat and green fruit, whose effect on the human frame must have been felt by the digestion more than by the brain.

On the other hand, the memory is improved by combing the hair, especially after sleep and exercise. Moderate joy and honest consolation are also good, not only for the memory, but for the intellect and conscience. Washing the head every four days in water in which sage and laurel leaves have been boiled is recommended, and a gentle walk after food is considered very serviceable.

Well, we have no objection to any of these remedies. They are harmless, at the worst; and if they leave the memory pretty much what it was before they were employed, they are none the less useful in other ways. At least, they are better than the fearful and wonderful system of mnemonics with which some students, by the advice of their "crammers," saturate themselves before an examination. Very ingenious things are mnemonics, very pretty and clever indeed to read; but when it comes to recollecting them, it is totally different. We have always found that if we could remember the fact that was to be remembered, we could recall the mnemonic that was to act as an "aid to memory;" but not otherwise.

